# Small nuclei identification with a hemispherical brain PET

**DOI:** 10.1186/s40658-022-00498-4

**Published:** 2022-10-08

**Authors:** Miwako Takahashi, Go Akamatsu, Yuma Iwao, Hideaki Tashima, Eiji Yoshida, Taiga Yamaya

**Affiliations:** Department of Advanced Nuclear Medicine Sciences, Institute for Quantum Medical Science, National Institutes for Quantum Science and Technology (QST), 4-9-1 Anagawa, Inage-ku, Chiba, 263-8555 Japan

**Keywords:** Brain PET, FDG, Healthy volunteer, Thalamus, Raphe nucleus

## Abstract

**Background:**

To confirm the performance of the first hemispherical positron emission tomography (PET) for the brain (Vrain) that we developed to visualise the small nuclei in the deep brain area, we compared ^18^F-fluorodeoxyglucose (FDG) brain images with whole-body PET images.

**Methods:**

Ten healthy male volunteers (aged 22–45 years) underwent a representative clinical whole-body PET, followed by Vrain each for 10 min. These two scans were initiated 30 min and 45 min after FDG injection (4.1 ± 0.5 MBq/kg), respectively. First, we visually identified the small nuclei and then compared their standardised uptake values (SUVs) with the participants’ age. Next, the SUVs of each brain region, which were determined by applying a volume-of-interest template for anatomically normalised PET images, were compared between the brain images with the Vrain and those with the whole-body PET images.

**Results:**

Small nuclei, such as the inferior colliculus, red nucleus, and substantia nigra, were more clearly visualised in Vrain than in whole-body PET. The anterior nucleus and dorsomedial nucleus in the thalamus and raphe nucleus in the brainstem were identified in Vrain but not in whole-body PET. The SUVs of the inferior colliculus and dentate gyrus in the cerebellum positively correlated with age (Spearman’s correlation coefficient *r* = 0.811, *p* = 0.004; *r* = 0.738, *p* = 0.015, respectively). The SUVs of Vrain were slightly higher in the mesial temporal and medial parietal lobes than those in whole-body PET.

**Conclusions:**

This was the first time that the raphe nuclei, anterior nuclei, and dorsomedial nuclei were successfully visualised using the first hemispherical brain PET.

***Trial registration *:**

Japan Registry of Clinical Trials, jRCTs032210086, Registered 13 May 2021, https://jrct.niph.go.jp/latest-detail/jRCTs032210086.

## Background

Small nuclei in the brainstem and thalamus interconnect widespread nervous regions through their specific neurotransmitters, modulating motor, emotional, and cognitive functions. For example, the loss of dopaminergic neurons in the substantia nigra causes motor dysfunction [1], and pathological changes in the raphe nucleus seem to be involved in non-cognitive symptoms in Alzheimer’s disease [2]. Recently, the specific nuclei in the thalamus have become the target of deep brain stimulation for refractory epilepsy treatment as well as Parkinson’s disease [3–5]. However, the neuronal activity of these small nuclei remains difficult to assess because of the lack of methods for evaluating their function in vivo. Positron emission tomography (PET) can be used to measure glucose metabolism as a neuronal function using ^18^F-fluorodeoxyglucose (FDG), an analogue of glucose, or other specific tracers; however, the performance of current PET systems has not reached the ability to evaluate these small nuclei.

One of the solutions to improve PET performance is to reduce the detector ring diameter to the smallest possible size for the brain because the larger diameter causes degradation in spatial resolution by angular deviation or photon non-collinearity, as well as a decrease in the sensitivity of photon detection. However, most clinical brain imaging is performed using whole-body PET systems with cylindrical detectors with diameters larger than approximately 700 mm [6–8]. The most intensively used brain-dedicated PET is the high-resolution research tomograph (HRRT, CTI-Siemens), which has detectors placed in an octagon with a gantry diameter of 350 mm, attaining high-quality specification of 2.2-mm spatial resolution. However, the localization of the small nuclei still relies on magnetic resonance imaging (MRI) information because the boundaries between nearby structures seem to be obscure on PET images [9, 10]. This implies that the values measured by PET are also affected by nearby structures that are close to each other.

To further improve PET performance, the ideal shape of the detector arrangement should be hemispherical to be as close to the brain as possible. To the best of our knowledge, no brain-dedicated PET with hemispherical shape has been reported for practical use. Thus, we developed a brain-dedicated PET system with a hemispherical detector arrangement with a diameter of 279 mm, attaining 245 ps time-of-flight (TOF) resolution [11, 12] and a spatial resolution of 3.1 mm. In this study, we performed brain FDG imaging in volunteers using the brain PET we developed, and we identified the small nuclei in the brainstem and thalamus and provided the standard uptake values (SUVs) of each region and their correlations with volunteers’ age.

## Methods

### Participants

Ten healthy male volunteers (mean age, 34.6 years; range 22–45 years) were recruited through our institute’s website participated in this study. All the participants were right-handed except one, non-smokers (past and present), and did not have psychiatric or neurologic disorders or a history of head trauma. MRI of each participant was performed on the same day as the PET scans to confirm the absence of abnormal findings on 3D-T1 weighted MR images (T1WI), T2WI, FLAIR, or MRA using 3-T tomography (Verio, Siemens Healthcare GmbH, Erlangen, Germany). This prospective study was performed in accordance with the Declaration of Helsinki and approved by the institutional review board of our institute’s hospital (CRB3180004). Informed consent was obtained from all the participants.

### FDG-PET protocol

**Fig. 1:**
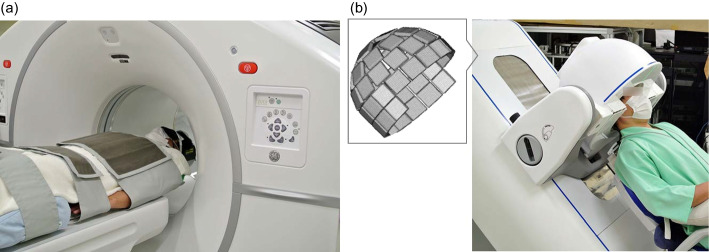
Photographs of whole-body PET (Discovery MI) (**a**) and hemispherical brain PET (Vrain) with a schematic view of the detector arrangement in the expanded view (**b**)

All participants fasted for at least 6 h prior to receiving their FDG injection and were confirmed to have a blood glucose level below 100 mg/dL. Each patient was administered 4.1 ± 0.5 MBq/kg (range 241.7–323.4 MBq) of FDG after resting for 5 min in the supine position on the bed of the whole-body PET in a quiet environment while closing their eyes and wearing an eye mask. Each participant maintained this resting status until the first PET scan was completed, which was started 30 min after the injection, using a representative current clinical whole-body PET/CT system (Discovery MI; GE Healthcare, Milwaukee, WI, USA) for 10 min. CT (120 kV, 200 mA) images were acquired immediately before the PET scan. Next, the patients were moved to another room on the same floor to undergo a Vrain scan, which was started 45 min after FDG injection and lasted 10 min. These two 10-min brain scans were performed with the participant’s head and chin fixed to the scanner’s headrest using dedicated bands. Vrain was approved by the Pharmaceuticals and Medical Devices Agency in Japan and was commercialised by ATOX Co., Ltd. (Minato-ku, Japan). Figure 1 shows one participant undergoing a brain PET scan with the whole-body PET system and with Vrain. In Vrain, the participants underwent the PET scan in the sitting position with the backrest of the chair reclined by 45°. The gantry of the Vrain can tilt to cover the head such that the angle of the Vrain’s field of view and the head are the same as whole-body PET.

The Vrain consists of 54 block detectors, 45 of which are arranged to form a hemisphere, whereas the other nine are placed to form a half-ring behind the neck. The inner ring diameter was 279 mm at the bottom ring, and the axial field of view (FOV) was 224 mm from the top detector to the bottom of the neck detectors. The detector block was composed of lutetium fine silicate crystals and a silicon photomultiplier with one-to-one coupling [11]. There were a total of 7,776 crystals. The dimensions of each crystal were 4.14 × 4.14 × 10 mm^3^. The spatial resolution was 3.1-mm at full width at half maximum (FWHM) at 1 cm, 3.6 mm at 10 cm from the centre of the FOV, and 3.0-mm FWHM at 1 cm from the centre at the base of the hemisphere. These spatial resolutions were calculated based on the data of ^22^Na point source images reconstructed by filtered back projection (FBP) according to the NEMA NU 2–2018 standards. The TOF resolution was 245 ps. Brain PET images were reconstructed using ordered-subset expectation–maximization (OSEM), including the TOF information, with four iterations and eight subsets, and were smoothed using a 3D Gaussian filter of 4 mm FWHM. The matrix size was 140 × 140 × 112 with a voxel size of 2.0 × 2.0 × 2.0 mm^3^. Attenuation correction was performed using CT images acquired with whole-body PET/CT by co-registering the PET and CT images for each participant.

Whole-body PET/CT (Discovery MI) consists of five 744-mm-diameter block detector rings and has an axial FOV of 250 mm [6]. There were a total of 24,480 crystals made of lutetium yttrium orthosilicate. The dimensions of each crystal were 3.95 × 5.3 × 25 mm^3^. The FBP FWHM spatial resolution was 4.3 mm at 1 cm from the centre of the FOV. The TOF resolution was reported as 381.7 ps [13]. PET images were reconstructed using OSEM, including TOF information with four iterations and 16 subsets, and were smoothed with a 3D Gaussian filter of 4 mm in FWHM. The matrix size was 192 × 192 × 89, and the voxel size was 2.73 × 2.73 × 2.78 mm^3^.

### Image evaluation

The voxel values of all PET images were converted to SUVs using the patient’s weight and FDG dose. For visual evaluation, the whole-body PET and Vrain images were anatomically co-registered to each subject’s MRI T1WI using an automatic procedure that maximises mutual information and re-sliced with a voxel size of 0.8 × 0.8 × 0.8 mm^3^ with trilinear interpolation equipped in PMOD (PMOD version 3.7; PMOD Technologies Ltd., Zurich, Switzerland). Visual inspection was then performed using the PMOD viewer. When the small nuclei were identified, a 4-mm-diameter spherical volume-of-interest (VOI) were manually placed to include the highest FDG accumulation of the structure, or two 4-mm-diameter spherical VOIs were placed continuously if the structure was larger than 4 mm diameter. The average SUV of these right- and left-side values was calculated and compared with the participants’ age.

Next, all PET images were anatomically normalised using transformation parameters, which were obtained through MRI normalization of each subject to the anatomical standard template provided by the PMOD. Then, an automated anatomical labelling atlas [14], which is a template VOI consisting of 116 VOIs, was applied to the anatomically normalised PET images. The SUVs of the following representative areas were selected and compared between whole-body PET and Vrain images: frontal lobe, mesial temporal lobe, lateral temporal lobe, medial parietal (posterior cingulate and precuneus) lobe, lateral parietal lobe, occipital lobe, and striatum.

### Statistics

Correlations between the SUVs of each region and the participants’ ages were tested using Spearman's rank correlation coefficient test. Comparisons of the SUVs of each region between whole-body PET and Vrain were performed using the Mann–Whitney U test. Statistical significance was set at two-tailed *p* < 0.05. SUVs of each region are represented as the average ± 2SD.

## Results

### Visual inspection

**Fig. 2:**
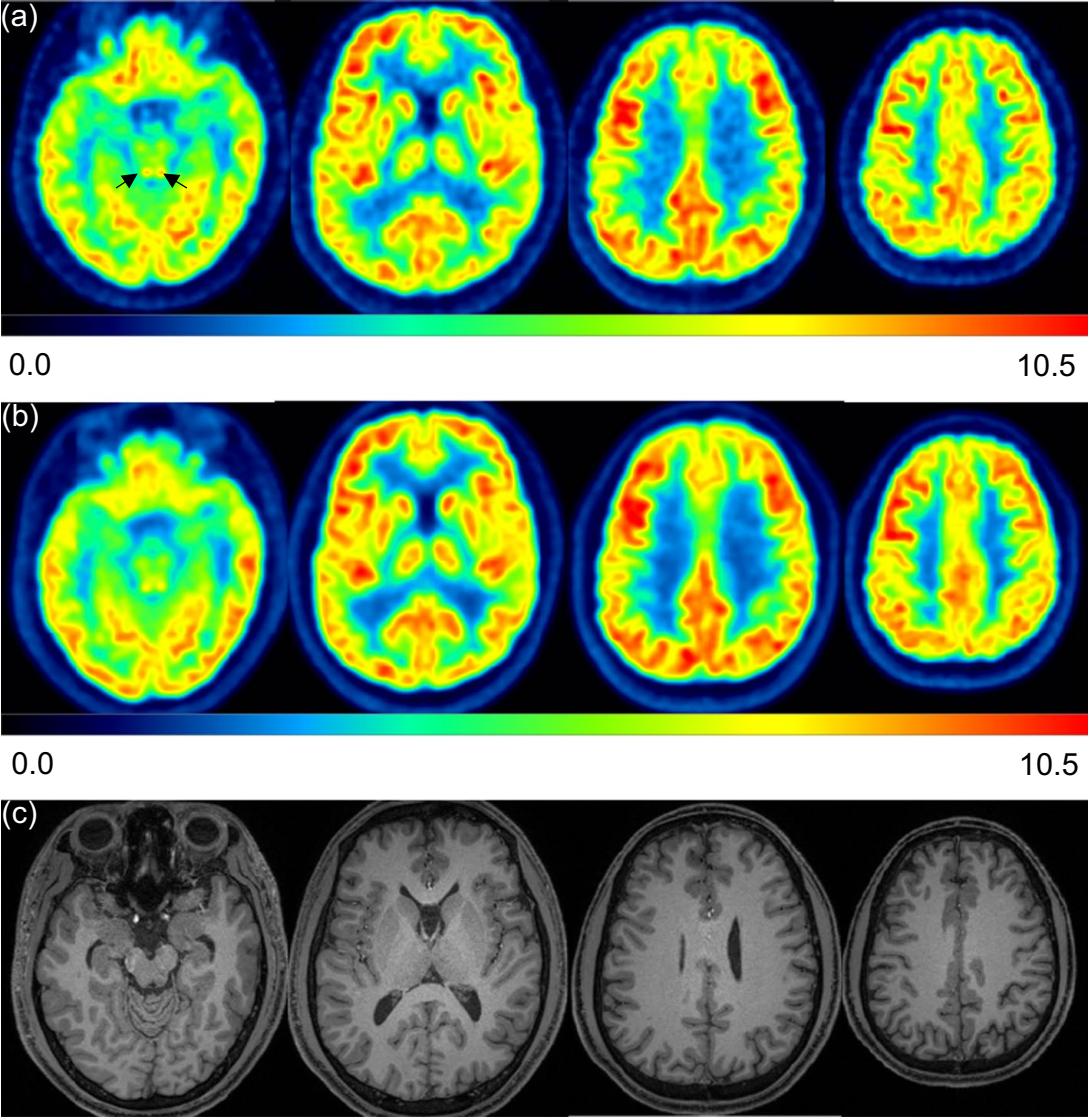
Representative images of FDG of the brain using Vrain (**a**) and whole-body PET (**b**). Axial images from the left are at the level of the inferior colliculus (arrows), basal ganglia, centrum semi-ovale, and fronto-parietal area. All PET images were co-registered to T1WI MRI (**c**). The SUV is indicated by the colour scale

**Fig. 3:**
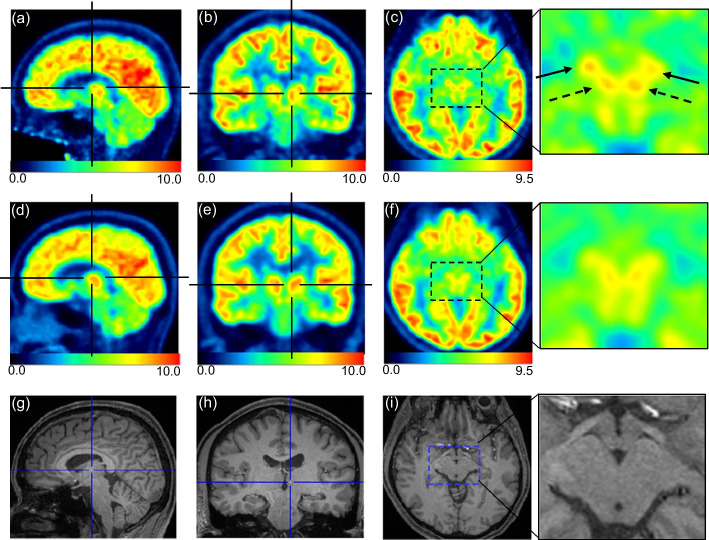
FDG-PET images using Vrain are shown at the top (**a**–**c**) and those using the whole-body PET are shown in the middle (**d**–**f**). Images **g**–**i** show the T1WI MRIs. The centre of each crossbar is placed on the anterior nucleus (**a**, **d**, **g**) or the dorsomedial nucleus (**b**, **e**, **h**) in the thalamus. These are clearly visualised using Vrain (**a**, **b**) but not using the whole-body PET (**d**, **e**). The substantia nigra (arrows) and red nucleus (dashed arrows) are visualised using Vrain (**c**) with an expanded scale for the squared area on the right, but each region is not visualised separately using the whole-body PET (**f**). The SUV is indicated by the colour scale

**Fig. 4:**
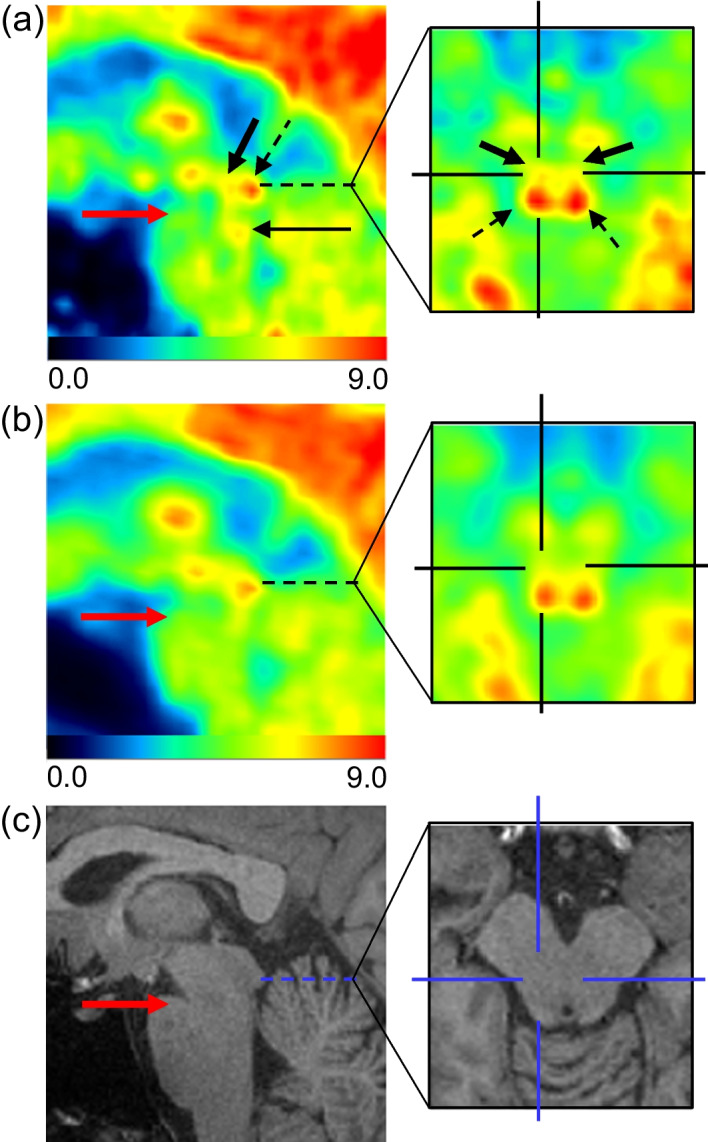
Sagittal views of the brainstem using Vrain (**a**), whole-body PET (**b**), and MRI (**c**). The right expanded images show the axial images at the level (dashed line in the sagittal image), which is 4 mm above the isthmus (red arrow). The midbrain part of the raphe nucleus (bold arrows) is depicted ventral to the inferior colliculus (dashed arrows) using Vrain (**a**), and the centre of the crossbar is placed at the right component of the raphe nucleus in the midbrain. The pontine part of the raphe nucleus is also visualised on sagittal view (black arrow) (**a**) at 2 mm below the isthmus (red arrow). The raphe nucleus is not visualised on the whole-body PET (**b**). The SUV is indicated by the colour scale

Representative images are shown in Fig. 2. Visualization of the cerebral cortices in the Vrain images was as good as or clearer than that of the whole-body PET images. The inferior colliculus in the midbrain was identified in both Vrain and whole-body PET images, but it was clearer in Vrain (Fig. 2a, left image with arrows). We also identified the anterior nucleus (Fig. 3a) and dorsomedial nucleus (Fig. 3b) in the Vrain images but not in the whole-body PET images. The red nucleus and substantia nigra in the Vrain were also identified for each (Fig. 3c), but these could not be separated in the whole-body PET images. The corresponding images of whole-body PET and MRI (T1WI) are shown in Fig. 3d–f and g–i, respectively. The raphe nucleus in the midbrain and pons was also identified in some participants using Vrain (Fig. 4a), but could not be identified using whole-body PET (Fig. 4b).

### Manual VOI analysis

**Fig. 5:**
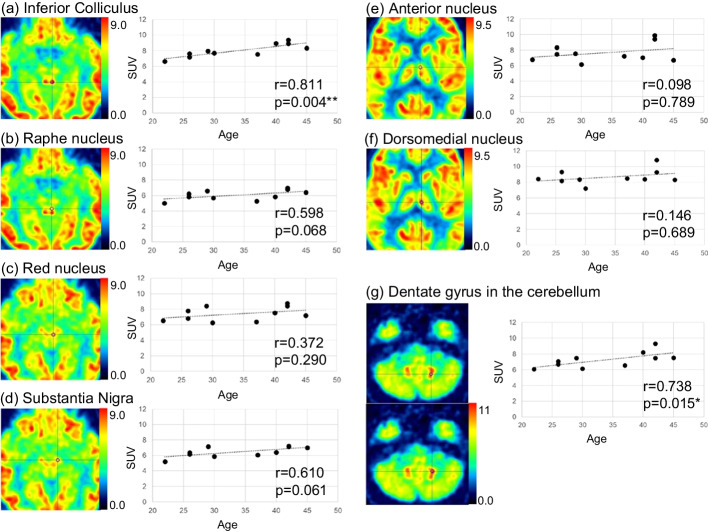
Representative images of 4-mm-diameter volume-of-interest (VOI) manually placed on each structure. The plots of the averaged standardised uptake values (SUV) of these right- and the left-side VOIs and ages are presented with the images

The inferior colliculus, red nuclei, and substantia nigra in the midbrain as well as the anterior nuclei and dorsomedial nuclei were identified. A 4-mm-diameter spherical VOI was then placed on the right and left to include the hottest area of each structure, and the average SUV of each region was calculated. Representative images with VOI placed on the left side and plots of the averaged SUVs of both sides and ages are shown in Fig. 5a–f. The raphe nuclei were most clearly visualised in participants in their 40’s. When the raphe nuclei were relatively obscure, we determined the location of the dorsal raphe nucleus at 4 mm above the isthmus and 4 mm ventral to the centre of the inferior colliculus, referring to the images of the 40’s age group and a study on the histological anatomy [15]. In addition, we placed four 4-mm-diameter spherical VOI in the dentate gyrus in the cerebellum (Fig. 5g) and then calculated the average SUV of the four VOIs and their correlation with age. A significant correlation between SUVs and age was found in the inferior colliculus and dentate gyrus in the cerebellum, but not in other structures. The average SUV of each region and the relationship between SUVs and age are summarised in Table 1.

**Table 1:** SUVs of each small nucleus visually identified in Vrain images and the relationship between the SUVs and age

	SUV in Vrain	Spearman’s correlation coefficient	*p* value^a^
Inferior Colliculus	8.0 ± 1.7	0.811	0.004*
Raphe nucleus	6.1 ± 1.3	0.598	0.068
Red nucleus	7.4 ± 1.8	0.372	0.290
Substantia Nigra	6.5 ± 1.3	0.610	0.061
Anterior nucleus	7.7 ± 2.4	0.098	0.789
Dorsomedial nucleus	8.7 ± 1.9	0.146	0.687
Dentate nucleus	7.3 ± 2.0	0.738	0.015*

SUVs are presented as the average ± 2SD

^a^*p* value represents the probability that the correlation could have occurred by chance

^*^p < 0.05

### Template VOI analysis

The average SUV of each region, based on the automated anatomical atlas [14], is shown in Table 2. The SUVs of the mesial temporal region and medial parietal region were statistically higher in Vrain than in whole-body PET by the Mann–Whitney U test, and other regions did not show any statistical differences.

**Table 2:** SUVs of each region based on the automated anatomical labelling atlas and the comparison of SUVs between whole-body PET and the Vrain

	Whole-body PET	Vrain	*p *value
Frontal lobe	7.2 ± 1.2	7.4 ± 1.4	0.280
Mesial Temporal	4.5 ± 0.7	4.7 ± 0.9	0.013*
Lateral Temporal	6.7 ± 1.3	6.9 ± 1.3	0.064
Medial Parietal	7.3 ± 1.3	7.6 ± 1.3	0.006*
Lateral Parietal	7.0 ± 1.3	7.1 ± 1.2	0.297
Occipital lobe	7.1 ± 1.4	7.2 ± 1.2	0.614
Striatum	6.3 ± 1.1	6.4 ± 1.4	0.219
Cerebellum	6.3 ± 0.8	6.2 ± 0.8	0.119
Numerical values; average ± 2SD			

SUVs are presented as the average ± 2SD

Automated anatomical labelling atlas[14]

^*^p < 0.05, Mann–Whitney U test

## Discussion

The hemispherical brain PET we developed, Vrain, enabled the visualization of the small nuclei in the brainstem and thalamus. To the best of our knowledge, this is the first time that such small nuclei in deep brain areas have been identified on FDG-PET images. In previous reports, glucose metabolism was calculated using brain-dedicated PET, but its localization was determined based on MRI [9, 10, 16]. Even with MRI, the components of the thalamus and raphe nucleus are difficult to visualise. Visualization of these small nuclei suggests that high-performance PET is achieved by Vrain because deep brain areas tend to be affected by common physical errors in the PET system, such as degradation of the detector sensitivity and angular deviation from annihilation photon collinearity. Accumulated evidence also suggests that these small nuclei may be altered in the early phase of neuronal degenerative disease [17–19], seem to be responsible for non-cognitive function [2], and become the target of neuronal diseases [3–5]. Therefore, high-performance brain PET imaging will be helpful in identifying early diagnosis, anatomical information of the treatment targets, and evaluation of neuronal function in the course of disease progression and responsiveness to treatments.

When we visually inspected the thalamus in the Vrain, the anterior and dorsomedial nucleus could be identified, and three more regions in the lateral area of the thalamus were also visualised but were relatively obscure and difficult to delineate consistently for all participants. Even in the histological anatomy, the parcellation of the thalamus remains controversial, but the intralaminar formation, such as dense feltwork fibres, divides the thalamus mainly into three parts: the anterior, medial, and lateral nuclear regions [20]. Therefore, we believe that the appearance of the thalamus in Vrain images follows this coarse separation, visualizing the anterior and dorsomedial nucleus. The lateral area is more complicated and has individual variations; therefore, this area may not be consistently visualised.

The neuronal morphology and cell density of the raphe nucleus have been investigated using postmortem Nissl-stained sections, revealing the nucleus located rostro-caudally in the central grey matter ventral to the cerebral aqueduct midbrain, from the level of the Edinger–Westphal nucleus to the level of the motor trigeminal nucleus in the pons [15, 21]. Baker et al. demonstrated that the number of neurons in the raphe nucleus was highest at 4–5 mm above the isthmus. On axial slices at that level, the nucleus histologically appears as a fountain-like shape composed of two wings bilaterally, with the size in the left–right direction at approximately 6 mm [15]. This fountain-like shape was also depicted in the Vrain images, as shown in Fig. 4a. Furthermore, localised FDG accumulation was also identified in the pons approximately 2 mm below the isthmus (Fig. 4a), corresponding to the location with the second-highest number of neurons in the raphe nucleus [15]. Therefore, we believe that these are visualizations of the midbrain and pontine parts of the raphe nucleus.

The SUVs of the inferior colliculus and dentate gyrus in the cerebellum were positively correlated with age. In this study, the raphe nucleus was more clearly visualised in volunteers in their 40 s than in those in their 20 s. These findings were consistent with a previous report, where the metabolic activity of the brainstem and cerebellum increased with age to approximately 35 years or one more decade [22–24]. These age-related increases in regional cerebral glucose metabolism from younger to older adulthood over adolescence suggest physiological brain maturational processes or compensation to maintain brain function, which is suspected to be associated with various age-related physiological changes or impairments.

We determined the start time of the PET scan based on the Alzheimer’s Disease Neuroimaging Initiative (ADNI) protocol, in which FDG-PET was acquired 30–60 min postinjection (https://adni.loni.usc.edu/methods/pet-analysis-method/pet-analysis/). Therefore, whole-body PET and Vrain were initiated 30 min and 45 min after injection, respectively. The time difference is one limitation of this study because during this time, the accumulation of FDG slightly increases in the brain grey matter as it approaches the plateau and almost reaches a plateau in the white matter [25]. The clearer visualization of the brain images with Vrain might be partially attributed to this time difference.

## Conclusion

We demonstrated that the raphe nuclei in the midbrain, and the anterior nuclei and dorsomedial nuclei in the thalamus were successfully visualised using Vrain, the first hemispherical brain PET we developed. These small nuclei were not identified in the representative clinical whole-body PET, which were also widely used in brain imaging. The substantia nigra and red nucleus were depicted separately in Vrain but not in whole-body PET. The small nuclei were clearly depicted so that we were able to calculate the SUVs for each, which will be helpful to investigate various neuropsychiatric disorders.

## Data Availability

Anonymized image datasets obtained in this study would be shared upon reasonable request to the corresponding and participating authors.
